# Printability and Cell Viability in Extrusion-Based Bioprinting from Experimental, Computational, and Machine Learning Views

**DOI:** 10.3390/jfb13020040

**Published:** 2022-04-10

**Authors:** Ali Malekpour, Xiongbiao Chen

**Affiliations:** 1 Department of Mechanical Engineering, College of Engineering, University of Saskatchewan, 57 Campus Drive, Saskatoon, SK S7N5A9, Canada; 2 Division of Biomedical Engineering, College of Engineering, University of Saskatchewan, 57 Campus Drive, Saskatoon, SK S7N5A9, Canada

**Keywords:** 3D bioprinting, extrusion, printability, cell viability, bioink, tissue engineering, machine learning

## Abstract

Extrusion bioprinting is an emerging technology to apply biomaterials precisely with living cells (referred to as bioink) layer by layer to create three-dimensional (3D) functional constructs for tissue engineering. Printability and cell viability are two critical issues in the extrusion bioprinting process; printability refers to the capacity to form and maintain reproducible 3D structure and cell viability characterizes the amount or percentage of survival cells during printing. Research reveals that both printability and cell viability can be affected by various parameters associated with the construct design, bioinks, and bioprinting process. This paper briefly reviews the literature with the aim to identify the affecting parameters and highlight the methods or strategies for rigorously determining or optimizing them for improved printability and cell viability. This paper presents the review and discussion mainly from experimental, computational, and machine learning (ML) views, given their promising in this field. It is envisioned that ML will be a powerful tool to advance bioprinting for tissue engineering.

## 1. Introduction

During the last decade, the demand for organ transplantation has increased all over the world because of rising success in post-transplant results. Unfortunately, inadequate organs for transplantation to meet existing demands have led to a huge organ shortage crisis [1]. Tissue engineering, as a combination of biology, engineering, and material science, is a promising field that can obviate this crisis by producing artificial tissue and organs [2]. Tissue engineering has advantages over other therapies (e.g., the use of drugs) because of its ability to provide a lasting solution to the problem of organ failure [3]. A scaffold is a porous construct used in tissue engineering to support cell growth and funcations. Several fabrication techniques have been developed and used to fabricate scaffolds, and in general, these techniques can be divided into conventional, electrospinning, and three-dimensional (3D) printing. Further, 3D printing can be categorized into inkjet, laser-assisted, and extrusion-based bioprinting [4]. Nowadays, extrusion-based bioprinter is one of the most popular techniques in biomedical applications because of its ability to print cells, and various biomaterials with a wide range of viscosity [4,5,6,7,8,9,10,11,12,13].

Biomaterials with living cells (or bioinks) or biomaterials without cells (inks) are printed to form constructs or scaffolds. To this end, the biomaterials must have such characteristics as (a) biocompatibility, (b) biomimicry, (c) mechanical integrity, (d) degradability, and (e) printability [14]. With 3D printing, it is expected that the printed scaffolds can mimic the structures, properties, and functions of particular organs for tissue engineering. Hence, the keys are the printing of scaffolds and their culture for maturation to functional constructs. For the printing of scaffolds, the printability of the chosen biomaterials must be considered before the printing process because of the differences seen between printed scaffolds and the designed ones [15,16]. Although incorporating cells with biomaterial is a promising advance in bioprinting, cells might be affected and even damaged during the printing process. Cell viability is another vital issue needed to be considered. For this, numerous studies have been conducted to fabricate biomimetic scaffolds with preserved cell viability in the extrusion-based bioprinting process. As the evaluation and optimization of printing parameters intended to achieve the improved printability and cell viability through trial and error was expensive and time consuming, computational methods came to play a role. Nowadays, with the advent of supercomputers, machine learning (ML) has depicted a new view for many fields of science and engineering, including biofabrication, with many promising results. It is envisioned that ML can accelerate bioprinting development. This paper aims to review printability and cell viability from experimental, computational, and machine learning perspective.

## 2. Experimental View

### 2.1. Printability

Generally, the printed construction may not be the same as the designed one. Sometimes, the printed structure collapses and cannot keep its stability. The printed biological product must mimic the architecture and shape of an organ, so the printing needs to be precise with high resolution and shape fidelity. The concept of printability refers to the ability to form a 3D structure with acceptable fidelity and integrity. Printability demonstrates its critical role when the tissue of an intricate organ such as the heart is required. In addition to function, geometry is very important [16,17]. There are various definitions for printability based on employed techniques for printing. Printability in the inkjet approach is recognized as the ability to generate well-defined droplets in the air. In laser-based printing, printability refers to the ability to produce a well-defined jet, form appropriate droplets, and deposit them onto the receiving substrate. For extrusion bioprinting, printability characterizes the capability to print continuous filaments with controllable diameter and defined morphology to form desired 3D structure [4,7,14].

#### 2.1.1. Evaluation of Printability

It is necessary to define indexes to characterize printed constructs in terms of printability. It is noted that a bioink needs to have appropriate shear thinning behaviour with low viscosity when being printed and recovered or high viscosity for stability after printing. To evaluate the printability of bioink, Paxton et al. [18] suggested a two-step method. First, this method focuses on screening ink formulation to assess filament formation and the ability to form 3D structure, and the second step is the rheological evaluation of inks to assess yield point, shear thinning, and recovery behaviour via shear stress ramp, shear viscosity, and recovery test, respectively [14,18]. There are different definitions for printability indexes. The most popular ones are reviewed as follows:

1. Extrudability: the minimum extrusion pressure required to print material at the desired flow rate [19];
2. Strand printability:
  This factor is used to compare printed strands dimeters with CAD-generated parameters strands. The expected strand diameter is [20,21]:
  (1) $$D_{S}=\sqrt{\frac{{4Q}_{t}}{{\pi V}_{n}}}$$
  where D_S_, V_n_, and Q_t_ are strands diameter, needle speed, and volumetric flow rate, respectively.
  So, needle speed affects the diameter of strands, and the below index is defined to compare printed strands with calculated ones:
  (2) $$\mathrm{Strand}\;\mathrm{printability}=\frac{\mathrm{Experimental}\;\mathrm{strand}\;\mathrm{diameter}}{D_{S}}*100$$
3. Integrity factor: this factor compares the thickness of printed scaffolds and designed ones
  (3) $$I=\frac{\mathrm{Scaffold}\;\mathrm{thickness}}{\mathrm{Control}\;\mathrm{thickness}}$$
4. Irregularity:
  In fact, this index is developed index of integrity in 3 dimensions. It compares the outer geometry of scaffolds with designed ones in X, Y, and Z directions [21]:
  (4) $$\mathrm{Irregularity}=\frac{\left|{\mathrm{Experimental}\;\mathrm{length}}_{X,Y,Z}\right|}{{\mathrm{Design}\;\mathrm{length}}_{X,Y,Z}}$$
5. Pore printability:
  In addition to printability indexes such as irregularity and integrity factors which are based on the outer geometry of scaffolds, the pore printability index focuses on internal geometry. This index is utilized to determine how printed pores matched the designed square ones in a scaffold [19,21]:
  (5) $$\mathrm{Pore}\;\mathrm{printability}=\frac{{\left(\mathrm{perimeter}\;\mathrm{pore}\right)}^{2}}{16*\mathrm{area}\;\mathrm{of}\;\mathrm{pore}}$$

#### 2.1.2. Effective Parameters on Printability

Various parameters play a key role in extrusion bioprintability that can be categorized into three groups: bioink properties, printing parameters, and design construct.

##### Bioink Properties

Bioinks may contain hydrogels, decellularized matrix components, tissue spheroids, cell pellets, or some advanced bioinks. The most popular class of biomaterials are hydrogels because of their capacity to provide a viable environment for the adhesion, growth, and proliferation of living cells. The main feature of hydrogels is their ability to absorb and retain large quantities of water. Hydrogels are non-Newtonian fluids with shear thinning and thixotropic behaviour, which are suited for the extrusion process. The shear thinning enables the shear force to align the random polymer chains in a favourable direction and makes them extrudable. Thixotropy, a time-dependent shear thinning behaviour, makes the hydrogels exhibit low viscosity during printing and regain stability after extrusion [22].

Rheological property is a critical key to the printability of hydrogels, and viscoelasticity is a conclusive factor in bioprinting. Printing low viscous materials leads to soft and watery structures; however, printing high viscous materials is difficult to extrude [23,24]. Examining the relationship between shear stress and viscosity under embedded shear rate provides applicable information for the printability of bioinks [25].

Differences between printing techniques have led to requiring different properties of hydrogels. For example, extrusion-based and inkjet-based systems utilize nozzles to deposit biomaterials. They require appropriate rheological properties, and the most important one is viscosity [26]. In fact, extrusion-based bioprinting is a modification of inkjet printing that can print uninterrupted strands instead of a single droplet. This printing technique can print almost all types of hydrogels with varying viscosity depending on the dispensing force system. Among extrusion approaches, a pneumatic system powered by pressurized air can support a wider range of viscosity but has difficulty precisely controlling deposited mass. Screw-based ones are cheaper but have problems with high viscous materials [2].

The viscosity of the hydrogel is an effective parameter for printability [27]; however, it cannot capture the complicated behaviour of bioink, and a high viscous hydrogel does not guarantee precise printing. Dynamic modulus, including storage modulus (G′) and loss modulus (G″), can describe the bioink behaviour better. The loss tangent (G″/G′) is another parameter that makes the biomaterial behave more like a solid or a liquid. A small loss tangent refers to a material with more solid behaviour, high stiffness, and good mechanical properties which can maintain its designed shape and require larger extrusion pressure. On the other hand, a large loss tangent represents highly fluid behaviour, easily extrudable material without adequate mechanical strength to hold the structure [19].

In addition to the flow behaviour of the bioink, surface tension is another physical property needed to be considered in extrusion bioprinting. At the liquid—air interface, surface tension appears because of the attraction force between liquid molecules rather than the attraction between liquid and air, thus affecting the profile or contour formed on the surface [28]. Surface tension is usually studied in inkjet-based printing, and its effect on printability is neglected in extrusion-based bioprinters.

Surface tension appears as a contact angle between two media and plays a key role in the printed strands and printing resolution. If the substrate has higher surface energy rather than the bioink surface tension, the ink will spread. In contrast, less substrate energy results in a higher contact angle and less spread [21,29].

##### Bioprinting Parameters

An extrusion bioprinter commonly consists of three main units: a container (e.g., syringe) containing biomaterials, a dispensing head that derives biomaterial out, and a receiving stage where the bioink is collected [4,22]. The parameters that control those three units and involve the printing process can affect accuracy and printing resolution (resolution is the smallest achievable diameters of strands [30,31]). In this matter, many investigations have reported that nozzle diameter, speed of the dispensing head, height of the nozzle, and flow rate of extruded bioink can affect the width and height of the printed strands [11,32,33,34]. The aforementioned variables must be balanced to achieve the desired printed strands with a uniform diameter and high pattern fidelity.

Dispensing pressure is the most important parameter in extrusion-based systems. Pressure should be large enough to overcome the ink surface tension for pushing the ink out of the nozzle. Under low pressure, hydrogels may not be driven to flow, while jetting would happen over high pressure. Obviously, there is a direct relationship between dispensing pressure, flow rate, and strands diameter [25,32,33,34].

**Figure 1:**
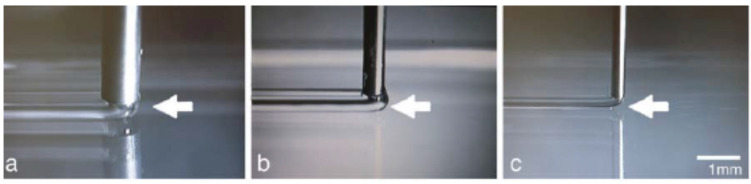
Images of printed hydrogels through (**a**) 20 ga, (**b**)25 ga, and (**c**) 30 ga needle (Effect of nozzle diameter and dispensing speed on strands diameter), Reprinted with permission form Ref. [35], 2011, John Wiley and Sons.

High flow rate, low needle linear speed, and high distance between the needle tip and the collector can result in thicker strands and a material bugle at the front edge of the needle (Figure 1b). In contrast, a low flow rate and a very slow needle movement cause a gap between the needle tip and stage (Figure 1c) [35].

The diameter of the needle is another important factor. Obviously, a lower diameter-needle can print a construction with high resolution (Figure 1), but clogging of the needle and high dispensing pressure are two limitations that should be considered. By increasing the concentration of the bioink, a needle with a higher diameter is preferred; however, needle temperature can improve the viscosity when high concentration bioinks are printed, especially for thermal-sensitive biomaterials [36].

During and after printing, to achieve the desired mechanical integrity and shape, the bioink must be solidified and cross-linked. Both materials and cross-linking agents should be prepared to reach appropriate viscosity, yield stress, and mechanical integrity for fast shear recovery [37,38,39,40]. Among cross-linking methods, adjusting the temperature of thermosensitive hydrogels [41] (e.g., gelatin), using cross-linking agents [42], and ultraviolet light [43] can be mentioned.

**Figure 2:**
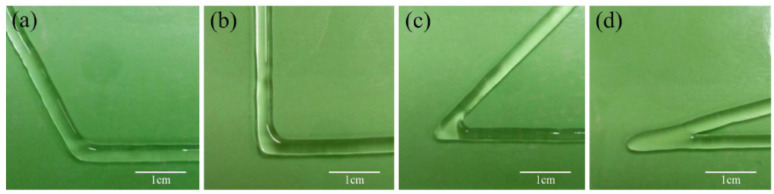
Overlap in corner printing: (**a**) obtuse angle printing; (**b**) right angle printing; (**c**) acute angle printing; (**d**) sharp angle printing [44].

There are some other key parameters such as distance between nozzle and substrate, substrate temperature and the angle of printing on the corners. As can be seen in Figure 2, the overlap problem in sharp angle during printing is a common issue that must be avoided. Printing resolution became worse in acute angles rather than obtuse ones at the same printing parameters, and it might cause nonuniform layer height in the 3D structures. There are two ways to avoid nonuniform extrusion. The first is to avoid the sharp angles in the printing, although sometimes sharp angles due to the complexity of the structure are inevitable. The second method is reducing the flow rate of bioink in this area to half or doubling the dispensing speed [44].

##### Construct Design

**Figure 3:**
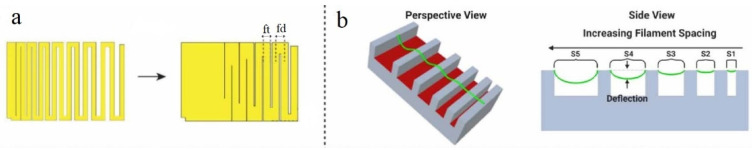
(**a**) Effect of spacing on filament fusion, fd—filament distance, ft—filament thickness; (**b**) filament over-hanger deflection, Reprinted with permission from Ref. [29], 2021, IOP Publishing, Ltd.

Filament spacing and filament orientation are two critical parameters in the matter of construct design that influence printability. Filament spacing affects pore sizes and the subsequent integrity and fidelity of the construct. A small filament spacing can lead to the fusion of adjacent filaments if the bioink has low viscosity and a small contact angle (Figure 3a). On the other hand, large spacing may result in a large over-hanger deflection (Figure 3b) [29].

The orientation of strands makes the configuration and inner pattern of the scaffolds and, subsequently, the porosity. The orientation of strands near the edge of the scaffold affects the amount of bioink deposited. To illustrate, an orientation of 45° requires less amount of bioink than 90° [21]

#### 2.1.3. Printability Improvement

The formulation of the ink with suitable rheological, biological, and mechanical properties are crucial aspects of the bioprinting process. The strategies for printability enhancement are related to the mentioned effective parameters (i.e., bioink properties, printing process, design). Table 1 lists the biomaterials commonly used in extrusion bioprinting owing to their appropriate rheological, mechanical, and biological properties. From these biomaterials, researchers have further synthesized composite biomaterials for their property synergy and improved printability [4].

There is a huge number of studies that evaluate various types of bioinks—a mixture of cells and biomaterials—to improve printability. As a good example, twelve types of hydrogels, including collagen, chitosan, fibrin, alginate, etc., were studied. Hydrogels were oriented over a 1 cm^2^ target printing area, and printing accuracy was calculated. MC-HA, chitosan, and chitosan–collagen gels were the most accurately printed ones because the relatively high viscosity of the solutions inhibited them from spreading out on the surface [80].

Alginate obtained from seaweed and algae forms a promising hydrogel for tissue engineering applications because of its inherent nature. Regardless of appropriate compatibility, the main disadvantage of alginate is the formation of unstable gels at lower concentrations because of low viscosity [47,81,82]. Although by varying the concentration and molecular weight of alginate, the viscosity could be increased, to overcome this problem and extrude filaments with well-defined morphology, gelatin can be added into the bioink [83]. Combining alginate with high molecular weight molecules like nanofibrillated cellulose also enhances the resolution of printed filaments [48]. Mouser et al. [84] added gellan into gelatin gum methacrylate (GelMA) to increase the viscosity and gelation speed of the hydrogel because printing GelMA on its own requires high concentration and precise control of the ink, nozzle, and receiving platform’s temperature.

To improve the printability of alginate, pre-cross-linked alginate with CaCl_2_ and a mixture of alginate and gelatin were printed and compared. The pre-cross-linked alginate was printed and formed a liquid-like scaffold with inconsistent pore diameters. In contrast, adding gelatin enhanced the printability of the ink significantly [42,83].

Adding additive biomaterials can change the rheology properties of hydrogels; for example, adding nanofibrillated cellulose (NFC) to alginate makes the bioink shear thinning with high fidelity [48]. GelMA-based bioink is another common biomaterial used for extrusion bioprinting because of its photo-cross-linking ability [85,86].

Using a supportive scaffold is a strategy to enhance shape fidelity. To illustrate, a printed PCL scaffold is used as a support for alginate scaffolds in the bioprinting of alginate [87,88,89]. These kinds of support can be sacrificial, especially for printing complex geometry [90]. Lee et al. [91], during printing poly-caprolactone (PCL) and cell-laden hydrogel, printed poly-ethylene-glycol (PEG) as a sacrificial layer to support the main structure. After finishing the process, PEG can be removed easily in an aqueous solution.

Among traditional implementation of printing techniques in an air medium, emerging technology is printing in a liquid bath or a hydrogel support bath medium. Printing in a support bath medium enables low viscous hydrogels to generate complex 3D structures. The limitations of printing in air, including clogging, gravity-induced structural collapse with weak interfacial strength, and the absence of a support structure, can be relieved using this approach. Specifically, low viscous materials minimize the occlusion of extrusion nozzles during printing within a support bath. The relationship between the diameter of strands and nozzle speed is similar to printing in the air. Increasing the flow rate by considering the constant filament’s diameter leads to higher nozzle speed and, subsequently, a less viscous environment for a shear thinning bath with large surface tension. This approach reduces irregularity and exhibits a smooth surface for the filaments [92,93].

**Figure 4:**
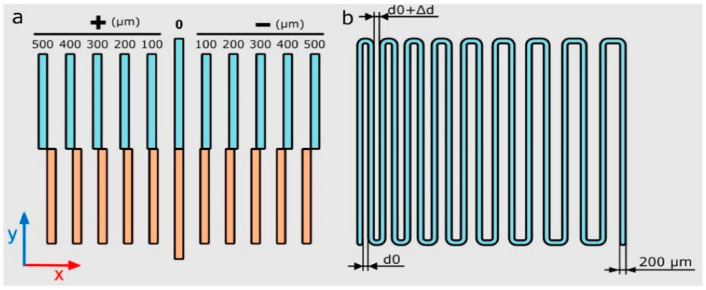
Schematic illustration of calibration models (**a**) xy offset of heads, (**b**) dispensing pressure and speed [94].

To print a complex construct with different materials for each part, utilizing a multihead bioprinter might be applicable. The calibration of the different heads plays a critical role during multihead bioprinting. For example, Sodupe-Ortega et al. suggested two models for calibration of a four-head printer depending on its application. In the first model, adjusting the printhead’s xy position with respect to each other, they printed straight lines, half with one printhead and half with the other. In the second model for optimizing dispensing pressure and speed, a continuous zigzag pattern was printed with an increasing distance between all lines (Figure 4) [94].

### 2.2. Cell Viability

The majority of the above studies were focused on the influence of various parameters on the printability of bioink; however, to achieve a successful 3D printing process, assessing different parameters’ effects on printability and cell viability at the same time is vital. The main advantage of extrusion bioprinting is the ability to incorporate cells with biomaterials, but it should be noted that cells are sensitive to environmental change. During the printing process, cells and biomaterials are extruded through a needle by force. Printing force can produce shear and extensional stresses. If these stresses exceed a certain threshold, they can breach cells membranes and damage them.

Shear stress has a critical influence on cell biology; to illustrate, shear stress enhances the maturation of some cells and increases stem cell differentiation [95,96]. In contrast, shear stress as a factor that damages cells is inevitable in any dispensing process and should be considered in printing progress [4]. Shear stress is the mechanical force that causes the shearing deformation of materials and cells along the plane parallel to the stress direction. Depending on cells’ sensitivity and the amount of shear stress experienced by cells, the cell damage can change up to almost 100% at high shear stress [97]. The shear stress is directly influenced by dispensing pressure, nozzle diameter, and viscosity of the bioink, especially when the needle diameter is reduced with the aim of promoting printing resolution [66,98,99,100,101,102,103].

Viscosity is a measurement of resistance to flow; highly viscous solutions can increase shear stress during extrusion and lead to the rupture of the cell membrane [104]. Printing high viscous bioinks requires high pressure, thus negatively affecting cell viability [97]. It was reported that dispensing pressure more significantly affects cell damage compared with nozzle diameter [105].

The nozzle diameter and type have direct effects on cell viability. Smaller nozzle diameter leads to a higher velocity gradient as well as higher shear stress and, consequently, higher cell damage [101]. Two types of needles, cylindrical and tapered, are commonly used in extrusion bioprinting. Because of their geometries, they have different effects; a tapered needle provides a higher flow rate than a cylindrical one under the same printing pressure. Indeed, for the same flow rate, lower printing pressure is required for tapered needles, thus preserving higher cell viability [106,107].

Nozzle and chamber temperatures are other effective factors in cell viability. By controlling the nozzle and chamber temperature, cell viability could be increased from 55.52% to 90% [108]. In addition to thermal damage, the time period or the duration of printing must be considered during bioprinting. Long bioprinting time can reduce cell viability after extrusion [109]. For improvement, hybrid polymer constructs made from mixing high and low melting temperatures were reported to recuse cell damage associated with temperature and bioprinting time [110]. The concentration of the bioink can also influence cell viability. It was reported that concentrated polymers have adverse effects on cell culture. For instance, there were more dead cells for a higher concentration of alginate [111].

Mathematical modelling is a tool to represent the bioprinting process as well as the influence of related parameters. In this regard, cell viability laws have been developed to depict the relationship between shear stress and cell viability. If shear stress is considered one of the main causes of cell damage, the following power–law function is used to describe the cell damage percentage [97]:

(6) $$I(\%)={C\tau }^{b}$$

where I is the percentage of cell damage, τ is the shear stress, and C and b are constants for given cells. This equation is developed further by considering the exposure time of cells to shear force [112]:

(7) $$I(\%)={\mathrm{Ct}}^{a}\tau^{b}$$

where I is the percentage of cell damage, τ is the shear stress, t is exposure time, C, a, and b are constants for given cells. This model has two drawbacks: First, this equation does not provide information about the probability distribution of cell damage with stress and exposure time and their correlation. Secondly, this equation is not applicable for a large range of stress or exposure time because, in this situation, the left side of the equation will be 100% [113]. Li et al. [112] improved this cell damage law by using a bivariate normal distribution function, and Nair et al. [105] also defined a law to predict damaged cells and dead ones separately.

## 3. Computational View

Three-dimensional printing potentially can revolutionize biofabrication engineering. The printability of the ink must be evaluated to achieve desired printed construct. Experimental evaluation of the ink printability is time-consuming and costly, especially when bioink with expensive and sensitive cells is printed [114]. In this endeavour, computational methods as powerful tools provide information to bridge gaps between knowledge when clinical testing is difficult, expensive, time-consuming, or even impossible [115].

### 3.1. Printability

Most of the printability simulations are in the field of inkjet bioprinting and droplet shapes. A study has been conducted to consider the influence of rheological properties of bioink on jetting behaviour during printing by an inkjet 3D printer. Computational fluid dynamic (CFD) has been employed through Flow 3D software to simulate rheological properties and droplet formation [116].

An investigation using the Continuum Surface Force (CSF) method for modelling surface tension and employing CFD-ACE+ commercial software investigated the effects of printing parameters on length tail, breaking time, and volume droplet [117]. Another study demonstrated the feasibility of using CFD to predict dependencies between printing parameters such as printer nozzle geometry, operation pressure, and printing speed in the extrusion-based bioprinting process. The rheological properties of the hydrogels were determined through different experimental methods and compared with data predicted by the computational model. The hydrogel viscosity was not predicted reliably because shear rates occurring within the printing tip were higher than the viscosity measured by the rheometer. CFD also was used to predict the relationship between resolution and printing speed. The predicted strand width depends on the contact angle between substrate and hydrogels [118].

**Figure 5:**
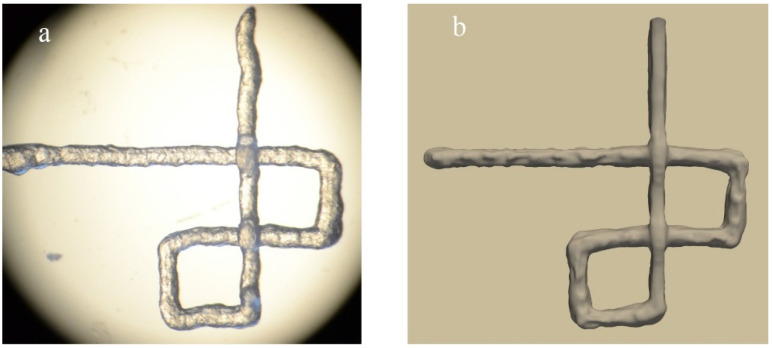
Visual comparison between (**a**) printed structure and (**b**) simulated structure [114].

The CFD via “OpenFOAM” software was employed to investigate the dependence between hydrogel mass flux, different needles geometry, and operating pressure. Power–law model was used for modelling Non-Newtonian hydrogels, and the VOF method tracked the interface between ink and air [118]. “IPS IBOFlow” commercial software also simulated printed strands. IBOFlow is an incompressible finite volume-based fluid flow solver based on Immersed Boundary Method. The rheology of the bioink was modelled by a linear PTT model, and the continuum surface force method [119] modelled the surface tension. The simulation had an appropriate agreement with experimental results (Figure 5) [114].

In the literature, because of the complexity of the simulation of printed strands, most studies have used commercial software and rarely discussed simulation methods. In this matter, a brief discussion about different methods to model multiphase fluid is found in the next part.

#### 3.1.1. Implementation

To evaluate the printability of the bioinks through computational methods, understanding the printed strands’ shape, in other words, the location of the interface between bioink and the air, is crucial. In fact, this simulation is a free surface fluid simulation. The term free surface is technically used to describe an interface between a liquid and a second medium that does not apply pressure gradient or shear stress. Free surface falls within the category of a multiphase flow problem. Some of the more popular techniques to track the interface are as follows [120]:

##### Surface Marker Techniques

This approach tracks the interface explicitly on a fixed mesh, marking the interface by connecting a set of massless particles. These markers are moved by the local advection velocity field, so their position (x^n^, y^n^, z^n^) can be obtained by integration from the initial position at time = 0:

(8) $$x^{n}{=x}^{0}+\int_{0}^{t}u\;\mathrm{dt}{,\;y}^{n}{=y}^{0}+\int_{0}^{t}v\;\mathrm{dt}{,\;z}^{n}{=z}^{0}+\int_{0}^{t}w\;\mathrm{dt}\;$$

where u, v, and w are the fluid velocity in the Eulerian mesh at the time-dependent location of each marker [121].

##### Surface-Fitted Method

In this method, instead of markers, a mesh surface is attached to the interface, and the position and curvature of the interface are calculated. The main advantages of this approach are (a) a reduction in computer storage occupied by the interface markers, (b) ensuring a sharp interface, and (c) avoidance of partially filled cells (every cell is occupied by a fluid). Since the mesh and the interface are moving together, the mesh automatically tracks the interface, and the mesh system conforms to the shape and structure of the interface. The weakness of this method is sensitivity to the mesh [121,122].

##### The Volume of Fluid (VOF)

**Figure 6:**
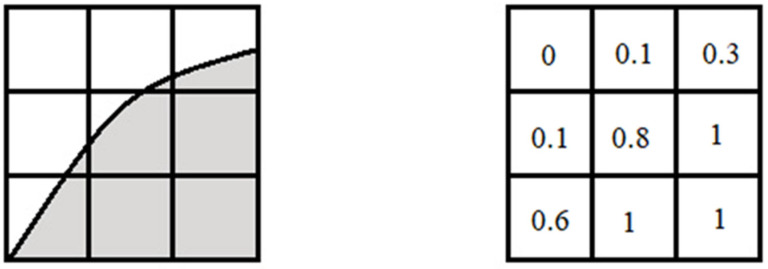
Value of colour function of cells based on the presence of different phases [123].

The volume of fluid is the most well-known method used to simulate multiphase flow. In this approach, the volume of fluid function, or colour function C, is used that represents the phase fraction. The volume fluid function where a cell is completely occupied by one phase (bioink/air) is unity and by another phase (air/bioink) is zero (Figure 6). According to this definition, the interface location is where the value of the colour function of the cells is between zero and unity. The main advantage of this approach is the accuracy in solving the interface equation without compromising the mass balance, and its weakness is difficulty with sharp interface and curvature. In addition, the implementation of this method in 3D cases is complicated.

As an example of showing how the colour function works in this method, density and viscosity can be calculated by the following relations:

(9) $$\begin{array}{l} {\rho (x,t)=\rho }_{1}{+(\rho }_{2}{-\rho }_{1})C\\ {\mu (x,t)=\mu }_{1}{+(\mu }_{2}{-\mu }_{1})C \end{array}$$

where subscripts 1 and 2 are related to phases one and two, respectively [123].

Obviously, by decreasing the interface thickness, the solution will be more accurate. At first, the initially prescribed topology of the interface is used to calculate the volume function of each cell. For this task, the location of volumes truncated by the interface for each cell is required. After providing velocity field by solvers through solving Navier–Stokes, the interface is reconstructed from the local volume fraction.

(10) $$\frac{\partial C}{\partial t}+u_{i}\frac{\partial C}{\partial x_{i}}=0$$

There are different methods to calculate volume fractions based on the topology of the interface [124]. The first method was Simple Line Interface Calculation (SLIC), presented by Noh [125], which represents the interface by a horizontal and vertical rectangle. Piecewise Linear Interface Calculation (PLIC) is another method that approximates the interface in each cell by an inclined line [126].

##### Level Set Method

The level set method is very close to VOF with a difference in representing the interface function. This class of interface captures the works based on the definition of a continuous level set function. Its magnitude is calculated through the distance between each cell and the interface. The sign of the function can be positive or negative based on the cell being in phase 1 or 2 (bioink and air) and zero value for the cells on the interface. The main advantages of this method compared with other techniques include the ability to predict sharp interface and attractive simplicity of mathematical formulation. Mass loss due to numerical errors can be noted as a disadvantage [127,128].

Surface tension is the dominant physical property that demonstrates the behaviour of the bioink after printing and must be known. Free surface molecules have higher energy than those in bulk. For a droplet of a liquid on a surface without external forces, the shape with the lowest surface energy and subsequently the lowest surface energy is a sphere [129]. When the surface of the separation is curved, the pressure near the surface is different in the two media. Surface pressure is proportional to the curvature k of the interface and the surface tension force directed toward the center of the curvature (higher pressure medium to lower pressure one) [119,129,130]. There are two ways to implement surface tension. One approach is applying surface tension as a boundary condition along the free surface. For a staggered mesh, interpolation is needed to ensure that the pressures at the free surface and center of cells are in correct relation. This technique is not appropriate because the cost of iterations at each time step is high, considering the time-step restriction due to stability issues. Another issue is the need for the exact location of the free surface for the next time step. However, some techniques such as VOF and level set can predict the interface location at the current time; this value is not known for the next time step [131]. To address the mentioned obstacle, Brackbill [119] has suggested a method called continuum surface force (CSF). In this method, instead of determining the exact location of the interface, surface tension is added to the Navier–Stokes Equations as a body force. By considering the interface between two inviscid fluids, the surface tension force can be written as below, where k (curvature) is taken as positive if the center of curvature is in the air.

(11) $$F_{\mathrm{SA}}{(x}_{s}{)=\sigma k(x}_{s}{)\hat{n}(x}_{s})$$

where σ is the surface tension coefficient of the bioink, k is the local curvature, $R_{1}^{-1}+R_{2}^{-1}$ with R_1_ and R_2_ being the principal radii of the surface curvature, and ${\;\hat{n}}_{}$ is the unit normal.

Finally, the surface tension force is calculated:

(12) $$F_{\mathrm{SV}}{=\sigma k\delta }_{s}n=\sigma \mathrm{kn}\frac{\left|\nabla \tilde{C}\right|}{\left[C\right]},{\;\delta }_{s}=\frac{\left|\nabla \tilde{C}\right|}{\left[C\right]},\;\kappa =-\nabla .\hat{n}$$

where the tilde and bracket denote smoothed value and the difference between the maximum and minimum value, respectively.

The continuum surface stress method (CSS) [132] is another method that, instead of computing the curvature of the interface and adding a force to the momentum equation, introduced surface tension as a correction to the momentum stress tensor. This approach is completely independent of the topology of the interface, and the main advantage of that is its ability to perform 3D simulations and looks promising for simulation of complex dynamics. The capillary pressure tensor *T* is defined:

(13) $$T=-\sigma \left(I-n⊗n\right)\delta_{s}$$

where I is the unit tensor and ⊗ is the tensor product operator

(14) $${\sigma k\delta }_{s}n=-\nabla .T$$

(15) $$F_{\mathrm{SV}}=-\nabla .T=\left[\sigma \left|\nabla C\right|I-\frac{\nabla C⊗\nabla C}{\left|\nabla C\right|}\right]$$

### 3.2. Cell Viability

**Figure 7:**
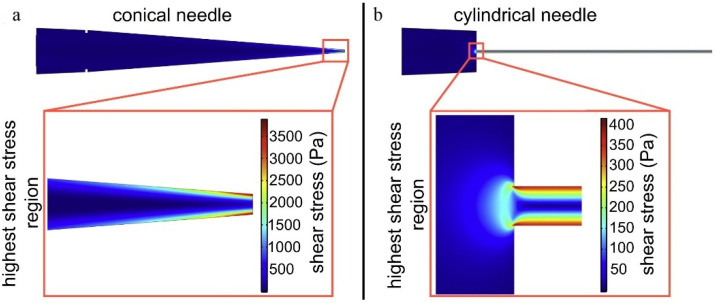
Heat map of the shear stress for (**a**) a conical and (**b**) cylindrical needle, Reprinted with permission from Ref. [36], 2014, Elsevier.

Experimental evidence shows the effects of nozzle geometry and bioink properties on cell viability; computational studies are helpful to gain more insight into this evidence [133]. Researchers defined a model based on the deformation and elongation of the cell membrane. The model connected (1) cell survival as a function of cell membrane elongation, (2) membrane elongation and cell droplet size, and (3) the substrate properties. The cell membrane may increase up to five percent approximately without cell death, and larger elongation can lead to rupture of the membrane [134]. A finite element simulation by COMSOL 4.0a software compared conical and cylindrical needle shapes on cell viability. The power–law equation was used to model non-Newtonian flow behaviour. The simulation showed that the highest shear stress was obtained for conical-shaped needles, but the cells withstand the stress just at a short region near the outlet of the needle. In cylindrical needles, a lower peak of shear stress occurs but in the long path of the needle (Figure 7) [36].

A sudden change in the geometry of the flow causes a significant change in linear velocity, and s, extensional flow occurs. Some studies employing CFD and using Fluent commercial software demonstrated that cells undergo dramatic stretching and deformation in the extensional flow, leading to cell damage [135,136]. Aguado et al. [137] showed that by reducing the diameter from 3.17 mm to 0.185 with the extensional flow, the cell is deformed. The cell viability from 89.1% (just linear shear occurs) decreased to 58.7% (extensional and linear shear flows occur); therefore, the main parameter that damages cells is extensional shear stress [113,137].

Romo et al. [138] employed CFD and OpenFoam software to simulate a series of experimental studies reported on the cell viability in a range of printing parameters such as pressure, nozzle shape (conical and cylindrical) and size, and material properties, to calculate maximum shear and optimize needle geometry. They found that the radius of the needle at the middle and outlet of conical needles play a key role in optimizing cell viability.

## 4. Machine Learning View

Machine learning is a promising technology that can optimize systems by using smarter and more effective use of materials, products, and services. Machine learning is a subset of artificial intelligence (AI) focusing on designing systems. This designing process works on learning and predictions based on previous experiences. In contrast to computer programing that relies on expert codes, machine learning techniques are trained to transform inputs to output via statistical relationships [139]. Humans are usually able to find a relationship between output (Y) and a set of input (X). When the input variables and outputs range from X_0_ to X_n_ and Y_0_ to Y_n_, humans will be overwhelmed by the complexity. Computer algorithms can guess and approximate functions among them, and that is the task of ML models.

**Figure 8:**
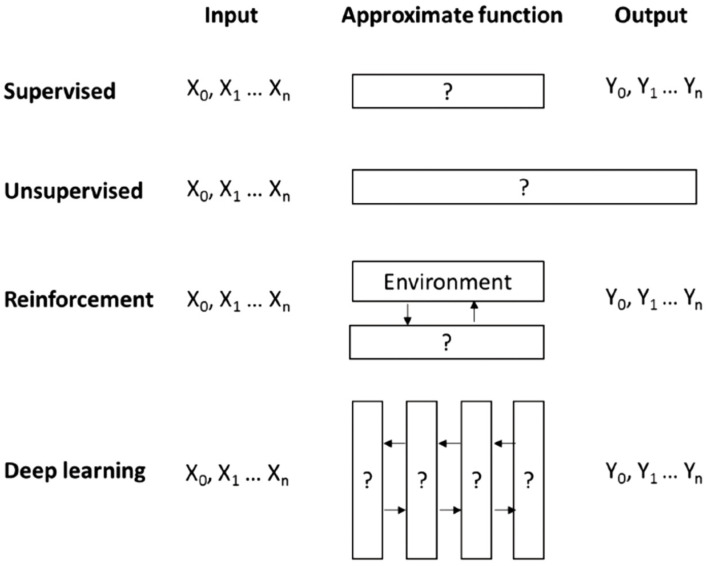
Example methods in machine learning [140].

The most common machine learning methods include supervised, unsupervised, reinforcement learning, and deep learning. In supervised ML, more inputs and outputs are available, and the approximate function will be found. In an unsupervised model, the output is not given, and the algorithm must find its own outputs as a pattern, a cluster, or a relationship in the data (X_0_, X_1_, … X_n_). Reinforced ML is another approach similar to the supervised one in which the inputs and output are given, and the algorithm must find the function between X and Y but through a dynamic interaction with another algorithm named environment. The environment rewards or punishes the main algorithm for making that more accurate. Deep learning is another method that employs a collection of algorithms with multiple hidden layers applied to a new dataset instead of dynamically adjusting the agent’s actions from the feedback. (Figure 8) [140].

Although machine learning has been used for improving 3D printing (additive manufacturing) in various ways, such as process optimization, manufacturing defect detection, and accuracy analysis, it has not been employed as much as it should in 3D bioprinting. Undoubtedly, it significantly affects the future development of this field [139,141].

### 4.1. Printability

Biofabrication works in the field of automated generation of functional biological products. Most of the techniques used in biofabrication were developed when additive manufacturing developed. Recently, ML was added to this field to cover key aspects impacting the biofabrication process efficiency directly. It would be valuable during material preparation, model designing, process optimization and monitoring. Developed algorithms can assist designers in choosing ideal printing orientation and material preparation, reduce design time, and consequently improve printability [142].

**Figure 9:**
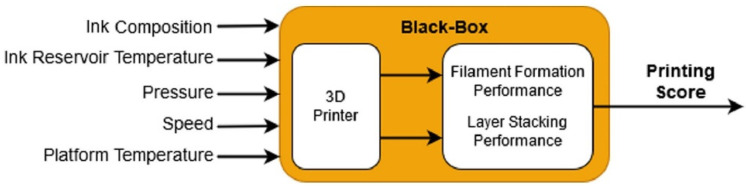
Optimization system based on machine learning, Reprinted with permission from Ref. [143], 2021, Elsevier.

**Figure 10:**
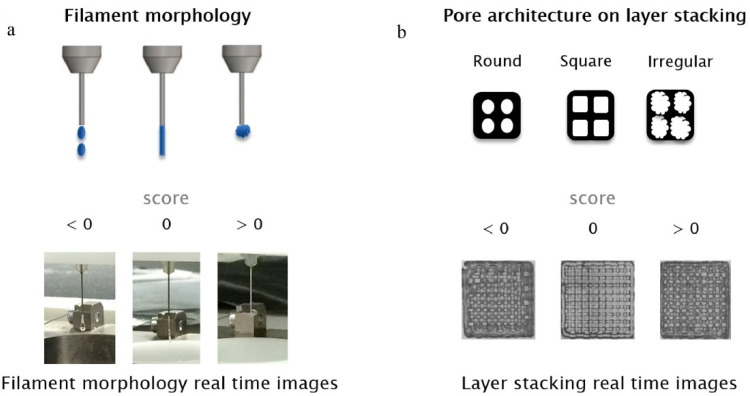
Evaluation of printability by two methods: (**a**) Filament morphology during printing and (**b**) Pore structure, Reprinted with permission from Ref. [143], 2021, Elsevier.

Raberu et al. [143] used machine learning as a novel method to evaluate printability quantitatively and optimize printing parameters. Ink concentration, temperature, driving pressure, needle speed, and platform temperature were considered as inputs (Figure 9). Printability was evaluated by printing scores based on two fundamental criteria: printing filament morphology during the extrusion process and pore architecture on later stacking (Figure 10).

Recognizing anomalies accurately in layer-by-layer bioprinted configurations is another potential task of ML in medical projects. A camera mounted at the side of the printhead captured images of each layer as raw data for the machine learning tool. Three major anomalies, including discontinuity (broken raster), irregularity (improper line width), and nonuniformity (unsmooth surface), were recognized in the first layer of printed structures. Machine learning could properly recognize the anomalies and optimize printing parameters based on them [144].

Shi et al. [145,146] employed a multiobjective optimization method and artificial neural network with computational fluid dynamics to simulate droplet formation and flow behaviour in drop-on-demand printing. Printing Silicone elastomer via freeform reversible embedding (FRE) is challenging due to depositing a Newtonian ink within a Bingham plastic support. To achieve this goal, hierarchical machine learning (HML) was employed, and the results showed that it is an effective tool to optimize printability factors [147]. Conev et al. [148] used an ML-based framework, printing conditions and printing parameters to predict the quality of print as “low quality” or “high quality”. Two methods were applied: a direct classification-based approach was used to train a classifier to distinguish between low and high printing quality, and a regression model was employed to approximate the values of a printing quality metric.

**Figure 11:**
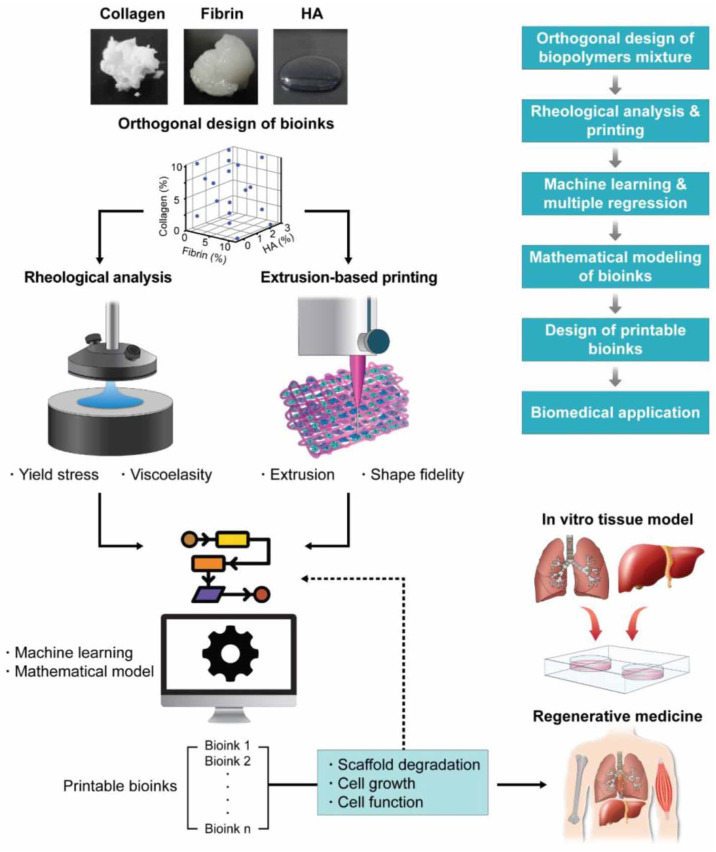
Schematic of bioink development based on mathematical modelling and machine learning, Reprinted with permission from Ref. [149], 2020, IOP Publishing, Ltd.

Another research utilized machine learning to predict the printability of various mixtures of collagen and fibrin. The rheological properties of inks were measured by a rheometer. Shape fidelity of inks was observed after printing and the data used by machine learning algorithms. As a result of machine learning analysis, the printable ink should have a high G’ for high fidelity and low τy for extrusion. A relationship was obtained to predict printable ink with high G’ and low τy. The Schematic of the process can be seen in Figure 11 [149].

### 4.2. Cell Viability

Despite the extensive experimental work carried out in extrusion-based bioprinting, a comprehensive view of individual and combined effective parameters on cell viability is not straightforward to achieve because of the various parameters. Different models of extrusion process have evolved from analytical models based on simplifying assumptions to using CFD to simulate complex flow behaviour and nozzles shape. Computational methods were used to optimize process parameters. Increasing computational power and rapid development of various algorithms are making data analysis techniques, such as machine learning, appropriate to address optimization challenges [138].

In the matter of employing ML to predict cell viability, little research has been reported. Reina-Romo et al. [138] developed an in silico framework to assess the effect of nozzle geometry, printing pressure, and material properties on the maximum shear stress as some of the main causes of cell mortality. They used CFD via OpenFoam software to simulate various shapes of nozzle and extrusion conditions; then, the Gaussian process was utilized to analyze the data and identify parameters affected by shear stress and related cell viability. Unlike ANOVA, the Gaussian process not only estimates the importance of individual parameters but also the influence of parameters on the outcome of a model is predicted.

Lack of data is the main challenge of the machine learning process, so available databases in the literature can be used as raw data for this kind of research. To illustrate, a dataset of 617 instances corresponding to a cell viability value and a dataset of 339 instances regarding a filament diameter in extrusion-based bioprinting systems were collected from the literature and used to train algorithms (ML). Regression-based and classification-based ML models were employed to predict cell viability and filament diameter for printing cell-laden alginate and gelatin bioinks. The results indicated that the classification-based ML can predict cell viability with an accuracy of 70%. More data gathering with a focus on the printing parameters can strengthen the database to provide higher accuracy [150].

Various kinds of algorithms can be used in ML. A streolithography-based bioprinting study used ML to develop a predictive cell viability model by considering four critical parameters, including UV intensity, UV exposure time, gelatin methacrylate concentration, and layer thickness. Four algorithms including neural networks [151] (an algorithm inspired by neurons in the biological brain), K-nearest neighbours [152] (a nonlinear algorithm working by averaging the output of k neighbours), ridge regression [153] (a continuous shrinkage algorithm that improves accuracy by adding a penalized term), and random forest [154] (a tree-based algorithm that builds a forest of uncorrelated regression trees) were combined to achieve an accurate model [155].

## 5. Conclusions and Future Work

Extrusion bioprinting has been widely employed to create cell-incorporated constructs for tissue engineering, and to this end, printability and cell viability are two critical issues that need to be addressed. The difference between printed constructs and designed ones represents a big challenge, limiting the progress to mimic native tissue organs for tissue engineering. Our review illustrated that varying characterizations of printability were presented in the literature and that many parameters that can affect the printability of constructs mainly include those related to bioink properties, printing process parameters, and construct design. The main advantage of extrusion bioprinting compared with other technologies rests in its ability to incorporate cells into the biomaterials for printing constructs, while the process-induced forces can cause damage to the incorporated cells (or cell viability)—another unneglectable issue in bioprinting. Extensional stress and shear stress are two major process-induced forces responsible for cell damage. Some parameters, such as needle type and size, bioink concentration, and dispensing pressure, play key roles in causing cell damage. With many promising investigations on printability and cell viability, this field is still in its early stage and rigorously determining the effective parameters remains challenging for future development. Determination or optimization process based on trial and error is expensive, difficult, time-consuming, and sometimes impossible; therefore, computational methods demonstrate themselves as powerful tools. To optimize the bioprinting process, many parameters are involved in an interdependent manner. Nowadays, machine learning, as a newfound technology, represents a new horizon in the field of 3D bioprinting. The combination of ML and bioprinting can accelerate the development of bioprinting and thus tissue engineering. Currently, the main challenge to moving forward with ML in bioprinting is the limited available data. As such, we urge that it is time to establish a worldwide data-sharing network in the field of bioprinting. Also, we note that because of different settings in terms of brands of bioprinters and software around the world, sharing data represents many issues to be addressed and that standardized data of each printer by using similar open-source software for all printers could be promising. It is envisioned that ML, though relatively new in the field of bioprinting, will revolutionize bioprinting and thus tissue engineering in the future.

## Figures and Tables

**Table 1:** Common biomaterials used in extrusion bioprinting.

Biomaterials	Advantages	Limits	Target Tissue/Application	Ref.
Alginate	• Water-soluble • High water-absorbing • Highly biocompatible • Rapid cross-linking	• Low viscosity • Lack of molecules adhesion	• Liver • Nerve • cartilage	[4,37,45,46,47,48]
Agarose	• Water-soluble • Responsive to temperature • Rapid gelation	• Poor cell attachment	• Cartilage	[46,49,50,51,52,53,54]
Gelatin	• Highly bioactive • Highly biocompatible • Responsive to temperature	• Poor mechanical properties	• Ovary • Nerve	[4,45,55,56,57]
Chitosan	• Antibacterial	• Slow gelation • Poor water solubility	• Bone • Cartilage • Drug delivery • Wound dressing	[58,59,60,61]
Collagen	• High cell attachment • Responsive to PH and temperature	• Slow gelation • Poor mechanical properties	• Skin • Nerve • Cartilage	[49,62,63,64]
Fibrin	• High cell adhesion • Highly bioactive	• Rapid degradation • Poor mechanical stability	• Nerve	[65,66]
Polycaprolactone (PCL)	• Low cost • Biodegradable polyester • Excellent rheological and viscoelastic properties upon heating	• Just for hard tissue • Extended degradation	• Bone • Drug delivery	[67,68]
Hyaluronic acid (HA)	• Water-soluble • Highly biocompatible • Good shear thinning properties	• Rapid degradation rate • Poor mechanical properties • Required modification for stable cross-linking	• Wound healing • Bone • Cartilage • Hearth • Nerve	[49,69,70,71,72]
Polyethylene glycol (PEG)	• Water-soluble High capacity for chemical modification	• Poor biodegradability • Poor cell attachment	• Would dressing • Bone	[73,74,75]
Polyurethane	• Highly biocompatible • Tunable dol–gel transition temperature	• Slow biodegradability • Poor cell attachment	• Cartilage • Drug delivery	[76,77,78,79]
